# Early Dysregulation of RNA Splicing and Translation Processes Are Key Markers from Mild Cognitive Impairment to Alzheimer’s Disease: An In Silico Transcriptomic Analysis

**DOI:** 10.3390/ijms26157303

**Published:** 2025-07-28

**Authors:** Simone D’Angiolini, Agnese Gugliandolo, Gabriella Calì, Luigi Chiricosta

**Affiliations:** IRCCS Centro Neurolesi “Bonino-Pulejo”, Contrada Casazza, Via Provinciale Palermo, 98124 Messina, Italy

**Keywords:** Alzheimer’s disease, mild cognitive impairment, splicing, ribosome, translation, transcriptomic data, peripheral blood

## Abstract

About one billion people worldwide are affected by neurologic disorders. Among the various neurologic disorders, one of the most common is Alzheimer’s disease (AD). AD is a neurodegenerative disorder that progressively affects cognitive functions, disrupting the daily lives of millions of individuals. Mild cognitive impairment (MCI) is often considered a prodromal stage of Alzheimer’s disease. In this article, we retrieved data from the online available dataset GSE63060, which includes transcriptomic data of 329 blood samples, of which there are 104 cognitively normal controls, 80 MCI patients, and 145 AD patients. We used transcriptomic data related to all three groups to perform an over-representation analysis of the gene ontologies followed by a network analysis. The aim of our study is to pinpoint alterations, detectable through a non-invasive method, in biological processes affected in MCI that persist during AD. Our goal is to uncover transcriptomic changes that could support earlier diagnosis and the development of more effective therapeutic strategies, starting from the early stages of the disease, to slow down or mitigate its progression. Our work provides a consistent picture of the transcriptomic unbalance of many genes strongly involved in ribosomal formation and biogenesis and splicing processes both in patients with MCI and with AD.

## 1. Introduction

Alzheimer’s disease (AD) is a neurodegenerative condition that alters neurocognitive functions and impairs the normal life of millions of people worldwide. Alzheimer’s affects between 50 and 75 percent of dementia patients. According to global statistical data, women are more likely than men to have AD, and the risk rises even further with age. The global prevalence of AD continues to rise in parallel with increased life expectancy, making it a major public health concern in aging populations [1]. The pathogenesis of AD is characterized by the accumulation of amyloid beta (Aβ) plaques and neurofibrillary tangles in the most affected areas of the brain: the medial temporal lobe and neocortical structures [2]. AD is a complex and multifactorial condition, influenced by a combination of genetic predisposition, environmental factors, and the natural process of aging. The etiology and the age at which symptoms initially manifest are two important characteristics that can be used to categorize the various types of AD. From the etiological perspective, AD can be divided into two major categories: the familial form (FAD) and the sporadic form (SAD). FAD represents 4–6% of all cases of AD. It is due to autosomal dominant inherited genetic mutations affecting genes involved in the production and metabolism of the amyloid-beta protein, such as amyloid precursor protein (APP), presenilin-1 (PSEN1), presenilin-2 (PSEN2), and apolipoprotein E (APOE). Mutations in the abovementioned genes exponentially increase the risk of developing the disease, often at a younger age and with a faster course [3,4]. The *APOE* gene has three common alleles, namely ε2, ε3, and ε4, that influence APOE structure and function, with implications for AD. The ε4 allele represents a strong genetic risk factor for AD, while ε2 is protective against AD [5]. The prevalence of ε4 carriers in MCI and AD patients is about 65% [6]. The SAD, on the other hand, is the most widespread and affects people who do not have a clear family history. Despite extensive research, the precise mechanisms underlying sporadic AD remain incompletely understood, though aging, inflammation, mitochondrial dysfunction, and impaired protein clearance pathways are considered key contributors [7,8]. Age of onset is also an important criterion for distinguishing forms of early onset, which occurs when symptoms appear before the age of 65; in these cases, the disease can be either familial or sporadic. In contrast, the late-onset form appears after age 65 and is by far the most common form of the disease, accounting for over 95% of cases. This form is almost always sporadic and closely linked to the biological processes of aging [9,10]. Beyond the age of onset, AD is also characterized by a progressive sequence of clinical stages, which reflect the level of cognitive and functional decline. It is possible to divide the course of the illness into three main stages. The first stage is a pre-clinical phase during which changes in the brain are already taking place but are not yet visible through obvious symptoms. A following stage is known as mild cognitive impairment (MCI), characterized by slight but detectable cognitive difficulties, which do not significantly compromise the autonomy of the person. The last stage is dementia, which manifests with more pronounced symptoms and is generally divided into mild, moderate, and severe stages, depending on the extent of cognitive decline and the impact on daily life [11]. As noted above, MCI is considered an intermediate stage between normal age-related cognitive decline and dementia, affecting up to 19% of adults over the age of 65 years [12]. The main three criteria used to diagnose MCI are the preservation of general cognitive and functional abilities, the absence of diagnosed dementia, and evidence of memory impairment that is a usual feature of AD [13]. In particular, individuals diagnosed with MCI are approximately ten times more likely to progress to AD compared to cognitively healthy peers; it is estimated 40–60% of them develop AD within 5 years of diagnosis [14,15]. Given this progression, early and accurate diagnosis is critical. The main methods for the diagnosis of AD are neuroimaging techniques including computed tomography, magnetic resonance imaging, positron emission tomography, and the clinical history of patients [16]. Recent advances in neurodegenerative research have also led to the development and standardization of biomarkers, including levels of Aβ and increased phosphorylated tau, which have significantly improved the diagnostic accuracy of AD. The use of these biomarkers unfortunately has some issues, including the invasive nature of the lumbar puncture necessary for the collection of cerebrospinal fluid, the high cost of biochemical analyses, and the increase in susceptibility to complications in elderly people or those with chronic comorbidities [17,18,19]. Considering the mentioned problems, current scientific research is increasingly moving toward identifying biomarkers in the blood that can be detected by minimally invasive methods. From this perspective, the present work aims to explore, through a comparative bioinformatics analysis, the alterations in the transcriptomic profiles of human blood samples. The study is based on a dataset of 329 samples, divided between patients with AD, those with MCI, and healthy controls (CTL). The aim of this study is to identify imbalances in biological processes that are compromised from the initial stages of the disease, with the purpose to find processes useful for earlier diagnosis and the development of more effective therapeutic strategies.

## 2. Results

In this study, we used the GSE63060 dataset obtained from the Gene Expression Omnibus (GEO) datasets repository, which includes 329 samples: 104 CTL, 80 MCI, and 145 AD patients. Data from 48,803 probes were available for each sample. Using the extracted data, we conducted two separate differential expression analyses.

### 2.1. Differential Expression Analysis

**Figure 1:**
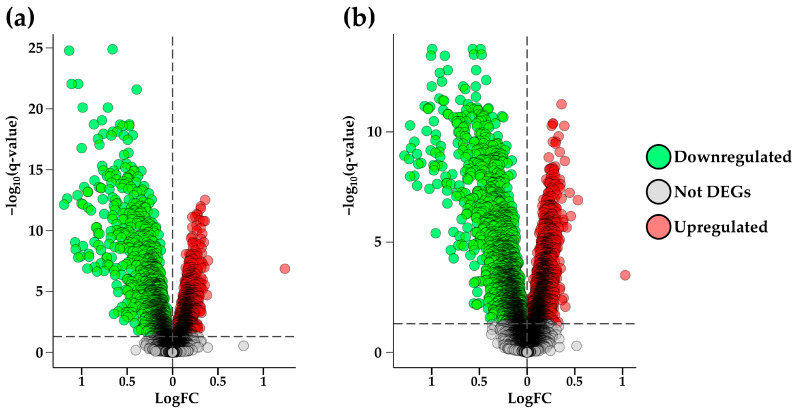
Volcano plots show all the probes inspected in the different comparisons. First plot (**a**) refers to the comparison CTLvsMCI, while the second plot (**b**) refers to the comparison CTLvsAD. In both plots, we report the LogFC in the *X* axis and the −log_10_ q-value in the *y* axis with a horizontal line that marks the statistical threshold set at 0.05.

The first comparison was performed between the CTL and AD groups (CTLvsAD) with the aim of identifying genes that are significantly deregulated in AD. This analysis was intended to highlight the differentially expressed genes (DEGs) potentially involved in the pathology. In the second analysis, we compared the CTL and MCI groups (CTLvsMCI) to uncover transcriptomic differences that may characterize the early stages of cognitive decline. This step aimed to identify early molecular alterations that precede the development of AD. The ultimate goal of our analysis was to overlap the results from both comparisons in order to identify DEGs that are already dysregulated in the MCI stage and remain significantly altered in the AD condition. This approach aims to highlight potential early biomarkers or key molecular players involved in the progression from MCI to AD. The comparison CTLvsAD resulted in 7300 transcripts with a *p*-value < 0.05. To minimize the false discovery rate (FDR), we applied the Benjamini–Hochberg post hoc correction and considered as statistically significant DEGs only those probes with a false discovery rate adjusted *p*-value (q-value) < 0.05. From the starting list of 7300 transcripts (with a *p*-value < 0.05), the number of significant ones, those with a q-value < 0.05, was reduced to 3280 after correction. From the starting lists of transcripts with a differential expression among the transcriptomic profiles we removed all those without an association to a gene name. The list of transcripts obtained from the comparison CTLvsAD was reduced from 3280 transcript to 3153 by removing 127 transcripts without a specific gene name association. At this point, we had a list of transcripts all associated with a gene name; so, we refer to them as DEGs. To obtain the final list of DEGs related to this comparison, we treated all the duplicated gene names maintaining those with a same trend of fold change (FC) across all the duplicated DEGs. After this last step of correction, we obtained the final list of 2760 DEGs, of which 1275 were downregulated, and 1485 were upregulated related to the comparison CTLvsAD. The upregulated genes were defined as DEGs exhibiting a statistically confirmed increase in expression in the AD group, whereas the downregulated genes displayed reduced expression levels in AD compared to the CTL. To obtain a large view on all the ontologies and processes that were altered in our transcriptomic profiles, we did not apply any filter based on the FC, and we included all the DEGs with statistically confirmed evidence. The same analytical workflow was applied to the second comparison CTLvsMCI that led us to identify 9623 transcripts with a *p*-value < 0.05. After applying the Benjamini–Hochberg correction, 4852 of them remained statistically significant (q-value < 0.05). The list of transcripts obtained from the comparison CTLvsMCI was reduced from 4852 transcripts to 4618, removing 234 transcripts without an associated gene name. In addition, for this comparison we performed an additional correction related to the duplicated gene names, reducing the list of DEGs from 4618 to 4035, of which 1895 were downregulated, and 2140 were upregulated. The complete set of *p*-values and q-values for each probe analyzed in the different comparisons is available in Supplementary Table S1. In Figure 1, we report the volcano plots that represents all the probes analyzed for both comparisons included in the analysis.

Our purpose is to highlight all the DEGs that begin to show transcriptomic alterations from the MCI condition that maintain this imbalance during the progression to AD.

### 2.2. DEGs Selection and Filtering

**Figure 2:**
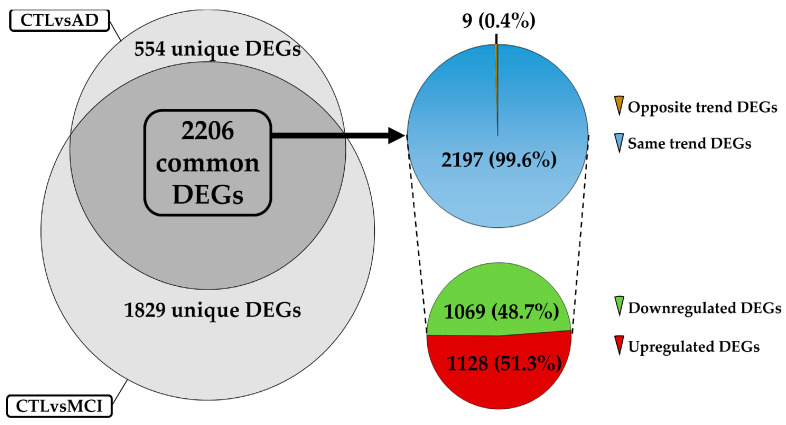
The left part of the figure contains a Eulero Venn graph that highlights the number of DEGs unique and common among the two different comparisons: CTLvsMCI and CTLvsAD. Among the 2206 shared DEGs, the first pie chart indicates how many DEGs assume the same expression trend among the DEGs. The pie chart in the lower part of the figure shows the distribution of up- and downregulated DEGs with same trend.

We merged the DEGs list of both comparisons to filter out the above-described DEGs, and the result was a list of 2206 shared DEGs. In contrast, 1829 were unique to the CTLvsMCI comparison, and 554 DEGs were unique to the CTLvsAD comparison. To be considered for the following analysis, we filtered the DEGs that had the same FC trend across the two comparisons, because our goal is to filter out those DEGs that can be monitored from the first insurgence of MCI that maintain that expression level imbalance in the confirmed AD condition. Among the previously mentioned list of 2206 DEGs, we observed that 2197 DEGs assumed same trend in both comparisons, and the remaining 9 assumed the opposite regulation trend among the different comparisons. These results are reported in Figure 2.

As shown in Figure 2, we obtained a list of 1128 upregulated DEGs and 1069 downregulated in both comparisons. For the following analysis, we focused our attention on this list of DEGs, excluding the remaining nine DEGs with opposite trend across the datasets inspected. We removed these because they are most likely not able to be considered as an early marker of pathology. The analysis continued by performing a gene ontology (GO) over-representation analysis (ORA) followed by a Benjamini–Hochberg post hoc correction to discover the ontologies more represented with statistical significance. For the abovementioned step, we needed to add the entrez gene ID to the gene symbol of our DEGs to improve the numbers of each gene detected for the enrichment. This step revealed eight DEGs (“LOC134997”, “LOC440354”, “LOC442454”, “LOC649946”, “LOC100008589”, “LOC153684”, “LOC255783”, “LOC338799”) without an associated entrez gene ID, and for this reason, we chose to remove them. From the new list of 2189, with all DEGs common to both comparisons with the same trend and with an associated Entrez ID, we discovered three DEGs with the same Entrez ID. Focusing on these DEGs, we discovered that each couple contained the most recent version of the gene name and an older version; considering that for all the DEGs, each gene had the same trend of FC, we removed the rows containing the older version of the gene name. Finally, we obtained the list of 2186 DEGs useful to perform the ORA analysis.

### 2.3. Over-Representation Analysis (ORA)

**Figure 3:**
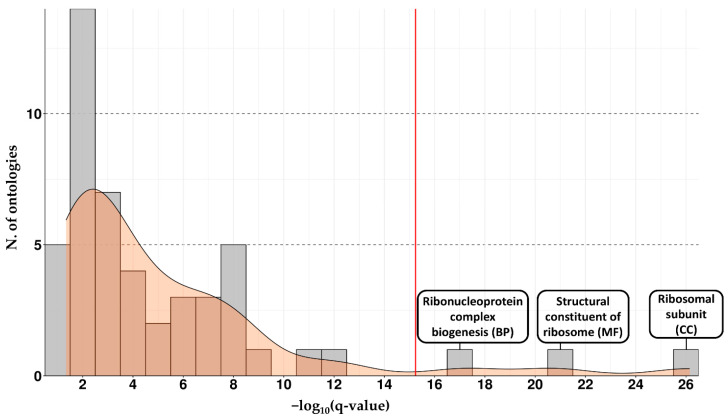
The bar plot shows the distribution of all the ontologies, where the y axis shows the number of ontologies for each range of q-value, and the x axis shows the q-value ranges. The red line indicates the 95th percentile of the distribution, and we report the description of the three ontologies over this threshold.

To be included in the list, each ontology must have an associated q-value < 0.05 and include at least 10 DEGs. These filters were applied to reduce the number of false positives and show the pathways with a sufficient number of DEGs. A total of 373 ontologies were enriched; the entire list with all the *p* and q-values and genes involved in each ontology is available in Supplementary Table S2. There was a high level of redundance among the ontology terms and, in order to reduce it, we applied the “simplify” function, included in the package “clusterProfiler”. Function “simplify” merges biologically related terms of the same ontology classes based on semantic similarity measures. Application of this function allowed us to condense the 373 ontologies by assimilating ontologies with overlapping biological meaning, and this provided a more concise and interpretable presentation of our results. After this step of analysis, we obtained 49 ontologies, available in Supplementary Table S3. In Figure 3, we report the number of ontologies for the different range of q-values to highlight those with the lowest.

As reported in Figure 3, three ontologies had a q-value above the 95th percentile: the biological process (BP) “ribonucleoprotein complex biogenesis”, the molecular function (MF) “structural constituent of ribosome”, and the cellular component (CC) “ribosomal subunit”. All these ontologies are strongly linked with processes involved in the ribosome that became the subject of the following analysis to observe how this process is altered in the MCI and AD conditions. Using all the DEGs involved in these three ontologies, we created a network to obtain a global view and highlighted all the interactions among the DEGs. The number of DEGs included in the ontologies was 121 for “ribonucleoprotein complex biogenesis”, 77 for “ribosomal subunit”, and 70 for “structural constituent of ribosome”; all of these are reported in Supplementary Table S4. In addition to these DEGs, in Supplementary Table S4, we provide the global list of 179 unique DEGs (151 downregulated and 24 upregulated) that includes all the DEGs from the three ontologies reported with the corresponding gene symbol in the STRING database. Many DEGs of the three starting lists are repeated across the different ontologies, and by removing the multiple DEGs and reporting them one time each, we obtained this final list of 175 DEGs that we used to build our network.

### 2.4. Network Analysis

**Figure 4:**
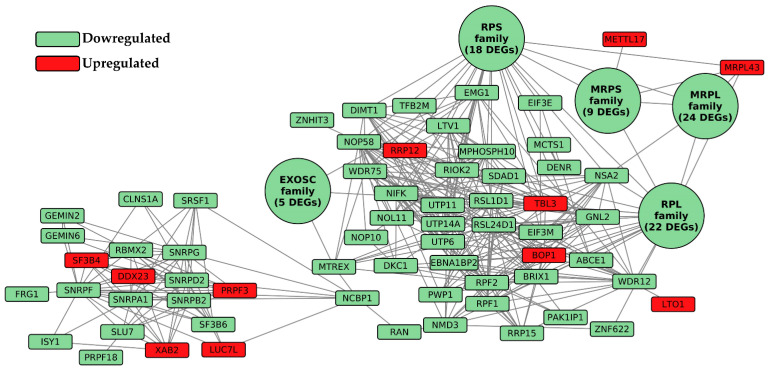
Network constructed using all the input DEGs. Downregulated DEGs are shown in green and upregulated ones in red. The large dots represent DEG families grouped together to improve clarity. All DEGs within the same family share the same fold change trend, which is reflected by the color of the dot.

The final list of DEGs was uploaded to STRING [20]. STRING is a database of protein interactions that provides different tools to obtain an interaction network. For our network, we selected the most stringent parameter (required score 0.9 and FDR stringency 1%) to select the strongest connection among our nodes. Starting from the original list of DEGs, we obtained a network of 171 nodes connected by 2081 bridges. Among these 171 nodes, we removed 28, because they were isolated nodes, without any connections. The results of the analysis of the network and all the information about the nodes are available in Supplementary Table S5. Considering that many DEGs with same trend of regulation were included in the same family of genes, we chose to collapse them into larger nodes to obtain a faster and easier view of the network. Figure 4 shows the network with the trend of FC, up- or downregulated, in the different comparisons.

In Figure 4, we can see a massive downregulation of the involved genes. In the large dots, we collapsed families of genes that shared the same trend of regulation (all were are downregulated). In the following table, we report all the DEGs collapsed into the five large dots in Figure 4.

The DEGs presented in both Figure 4 and Table 1 are further discussed in the following section.

## 3. Discussion

AD is a progressive neurodegenerative disease characterized by severe cognitive decline, while MCI represents a state of transition between normal aging and dementia with a high risk of progression to AD [21]. Around 40–60% of individuals affected by MCI will develop AD within five years [22]. The detection of certain biomarkers, including Aβ and tau, is essential for an accurate diagnosis of AD. Nowadays, these biomarkers are detected in cerebrospinal fluid, obtained through an uncomfortable and invasive lumbar puncture. In addition, neuroimaging techniques can help in AD diagnosis, but their high costs and low availability make them less advantageous. For these reasons, there is an urgent need to develop less invasive and easily available methods for AD diagnosis. In this context, blood biomarkers represent a promising approach. Blood biomarkers may be helpful for the early detection and diagnosis of AD, increasing the possibility of early intervention. In addition, a blood test may also support drug development, in order to identify and easily determine the effectiveness of the therapies. In this context, in our study, we found a transcriptomic profile associated with MCI and AD in blood for a non-invasive diagnosis. Moreover, we demonstrated that these transcriptomic alterations are detected in MCI patients, indicating that this method can be applied also for an early diagnosis, which is fundamental for early intervention. In this context, our study investigated transcriptomic profiles from 329 blood samples, divided between patients with MCI, AD, and CTL, to discover the impaired biological processes that could support earlier diagnosis and the development of more effective therapeutic strategies. Our analysis revealed a group of dysregulated genes mainly involved in ribosomal biogenesis and in processes such as the synthesis and regulation of rRNA, the formation of ribosomal structure, the nuclear export of ribosomal components, and the regulation of translation. Ribosome biogenesis occurs mainly in the nucleolus where the pre-rRNA, in association with proteins, forms the 90 S or Small Subunit (SSU) processome, enabling rRNA processing. Mature rRNAs are then assembled with a ribosomal protein into 40 S and 60 S subunits, which are exported as functional 80 S ribosomes [23,24]. Our study revealed a downregulation of several key genes involved in SSU biogenesis, including *UTP6*, *UTP11*, *UTP14A*, *NOL11*, and *WDR75*. In particular, *NOL11* and *WDR75* silencing has been shown to disrupt nucleolar organization and rRNA processing [25,26]. In parallel, we observed an upregulation of *TBL3*, a less-characterized gene known to be part of the SSU complex [27]. There are no literature data regarding the upregulation of *TBL3*; however, it is possible to hypothesize that the elevated expression may represent a compensatory response triggered by the downregulation of key genes involved in ribosome biogenesis. We also observed the reduced expression of several genes that encode for RNA-binding proteins essential for SSU formation, including *RPF1*, *RPF2*, *BRX1*, *ZNF622*, *NIFK*, *PWP1*, and *MPHOSPH10*. These factors are involved in pre-rRNA processing (BRX1, ZNF622) [28,29], subunit assembly (RPF1, RPF2) [30], and rRNA cleavage (MPHOSPH10) [31]. Additionally, the downregulation of genes that encode for core snoRNP components (*NOP58*, *NOP10*, *DKC1*) and methyltransferases (*EMG1*, *DIMT1*) [32] suggests impaired rRNA modification and maturation. The reduced expression of *ZNHIT3*, which encodes for a key factor in snoRNP assembly [33], further supports the concept of a global defect in the rRNA process and ribosome biogenesis in our model. The 60 S subunit biogenesis involves the PeBoW complex, formed by PES1, BOP1, and WDR12. In our study, we observed a downregulation of *WDR12* and an upregulation of *BOP1*. WDR12 plays a role in pre-rRNA processing and cell proliferation, and studies have shown that deletion of specific domains impairs these processes [34]. This imbalance in PeBoW components may compromise normal 60 S subunit maturation. The alteration in the initial processes of ribosome biogenesis involves a cascade event, also determining the dysregulation of cytoplasmic and mitochondrial assembly factors necessary for ribosome maturation. In this context, our analysis revealed a downregulation of the genes related to ribosome assembly factors: *EBNA1BP2*, *SDAD1*, *NSA2*, and *RRP15*. Although no specific studies are currently available that characterize in detail *EBNA1BP2*, *SDAD1*, *NSA2* and *RRP15*, which are assumed to have a role in the processing of pre-rRNA and assembly of ribosomal precursors, their downregulation could indicate an interruption in the production of ribosomes, probably as a response to cellular stress. We also observed an overexpression of the mitochondrial assembly factor METTL17, known for its role in the correct assembly of the mitochondrial ribosome, essential for mitochondrial protein synthesis and mitochondrial respiration [35]. This overexpression may reflect an attempt to maintain energy homeostasis in these cellular stress conditions. Our analysis shows also a significant downregulation of the genes related to RPL (ribosome proteins of large subunit) and RPS (ribosome proteins of small subunit) families, as shown in Table 1. The downregulation of these genes indicates a reduced ability to assemble functional ribosomes resulting in a possible decreased protein synthesis. The results observed are in line with the previous evidence that indicates a dysregulation of several ribosomal proteins in hippocampal samples from AD patients including a significant reduction in RPL30, RPL34 and RPL4, suggesting a possible link between ribosomal dysfunction and neurodegeneration [36]. Our study also revealed a significant downregulation of many genes encoding for mitochondrial ribosomal proteins of both the small subunit and the large subunit of the mitoribosome, MRPS and MRPL, respectively, as reported in Table 1. The reduced expression of mitochondrial ribosome proteins could impair the synthesis of mitochondrial respiratory chain proteins, promoting mitochondrial dysfunction and oxidative stress, which represent the main pathological mechanisms involved in neurodegenerative diseases [37]. Mitochondrial mRNA transcription and translation are tightly regulated processes. In this context, our study highlights the lower expression of TFB2M, a key factor in mitochondrial transcription [38], and ABECE1 which also plays a role in mitochondrial translation quality control [39]. We also observed an upregulation of *LTO1*. Recent studies associate an increase in the expression of LTO1 with a compensatory response to oxidative stress [40]. Furthermore, its unbalanced expression may also impair the assembly of the 4Fe-4S complex [41,42], thereby exacerbating mitochondrial dysfunction. Our analysis reveals an overall disruption of ribosomal subunit maturation and export, with significant downregulation of key regulatory genes such as *LTV1*, *GNL2*, *NMD3*, *PAK1IP1*, *RPS24D1*, and *RSL1D1*. The altered regulation of *GNL2* and *NMD3* is associated with the defective export of the 60 S subunit [43], while downregulation of *LTV1* and *RIOK2* suggests a block in the final maturation steps of the 40 S subunit [44], further supporting the global impairment of ribosome biogenesis. Another gene predominantly studied in yeast and found upregulated in our study is *RRP12*, which plays a role in pre-40 S subunit export [45]. Evidence of its induction under nucleolar stress [46] suggests a potential compensatory mechanism in response to impaired ribosome biogenesis, but its function in humans remains poorly defined. The impairment of the ribosomal assembly and export process leads to a reduced expression of translation initiation and reinitiation factors *EIF3E*, *EIF3M*, *DENR*, and *MCTS1*. eIF3e and eIF3m factors are essential to form the pre-initiation complex 43 S, helping mRNA recognition and preventing premature binding of the ribosomal subunit by ensuring the correct start of translation, while the DENR-MCTS1 is involved in translation reinitiation [47,48]. The low expression of eIF3 has also been observed in hippocampus samples from patients with AD, suggesting a correlation between altered proteins’ synthesis and progression of the disease [49]. In protein synthesis, a key role is played by the spliceosome, a ribonucleoprotein complex formed by numerous snRNPs and several accessory proteins, responsible for the maturation of messenger RNA through the process of splicing, which consists of the removal of introns and the correct assembly of exons [50]. Our analysis showed a marked downregulation of many spliceosome-related genes, including structural components (*SNRPG*, *SNRPD2*, *SNRPF*, *SNRPA1*, *SNRPB2*) [50], catalytic factors (*PRPF18*, *ISY1*, *SLU7*) [51], regulatory elements (*RBMX2*, *SRSFSF1*, *SF3B6*) [52], and genes involved in assembly snRNP (*CLNS1A*, *GEMIN6*, *GEMIN2*) [53], as well as general support for splicing (*FRG1*) [54]. These genes contribute to key stages such as junction site recognition, intron removal, and spliceosome assembly. In contrast, we observed the upregulation of *DDX23*, *PRPF3*, *LUC7L*, *SF3B4*, and *XAB2*, which are involved in spliceosome assembly, splice site recognition, and regulation between splicing and translation [55,56]. Another gene downregulated is *NCBP1*, a core component of the cap-binding complex (CBC), which binds to the 5′-cap of pre-mRNAs co-transcriptionally and regulates splicing, maturation, export, and translation [57]. Its depletion has been shown to impair spliceosome assembly [58]. Spliceosome dysfunction, evidenced by the downregulation and upregulation of numerous genes involved in complex assembly and regulation, can impair the efficiency of the pre-mRNA splicing process. The alteration of the splicing leads the activation of the exosome, a highly conserved multiprotein assembly complex that plays a crucial role in eliminating defective RNA and controlling RNA quality [59]. In our study, we observed a downregulation of genes coding for the exosome family reported in Table 1. These genes encode structural components of the Exo-9 catalytic core of the nuclear exosome complex, which is essential for RNA metabolism. In the nucleus, it processes stable RNAs (rRNA, snRNA, snoRNA) and degrades aberrant or incomplete transcripts. In the cytoplasm, it regulates mRNA turnover, controlling RNA quality and availability for translation [59]. We also observed downregulation of *MTREX* (Mtr4), a key component of the Nuclear Exosome Targeting (NEXT) complex. The NEXT complex guides nuclear exosomes in degrading aberrant and noncoding RNAs [60], and its impairment may compromise RNA surveillance and quality control mechanisms. Nucleocytoplasmic transport is essential for regulating protein synthesis. In our study, the downregulation of *RAN*, which encodes a GTPase critical for nuclear import and export of proteins and RNA, suggests a potential disruption of these processes. These altered processes could lead to misprocessing of mRNAs or accumulation of transcripts resulting in the production of aberrant or nonfunctional protein isoforms [61]. Table 2 provides an overview of some of the most relevant genes discussed in the text, along with their proposed roles in neurodegenerative processes.

Our results outline a consistent picture of nucleolar stress in MCI and AD, marked by altered ribosomal biogenesis and splicing, leading to reduced protein synthesis, mitochondrial dysfunction, and interrupted proteostasis with probably accumulation of misfolded proteins or a reducted number of functional proteins, known as key factors in neuronal decline [49,68]. Unfortunately, to the best of our knowledge, there are not available online datasets, reflecting our inclusion criteria, that also collect a multiomics characterization of the samples. Although our dataset includes basic demographic information such as age, sex, and ethnicity, it lacks more specific clinical and genetic variables, such as *APOE* genotype and age at disease onset. Inclusion of different omics information could offer additional context for interpreting inter-individual variability and supporting stratified analyses.

## 4. Materials and Methods

### 4.1. Dataset Selection

We performed systematic research across the public databases ArrayExpress [69] and GEO [70] to select the datasets with respect to our inclusion criteria. We performed the research on 10 May 2025. Our research used the keywords “Blood”, “Alzheimer” and “MCI”. We filtered out all the datasets that contained data obtained using microarray or RNA-seq technologies, necessary to perform a transcriptomic analysis. To be chosen for the analysis, the datasets needed to incorporate a large number of HC, MCI, and AD samples with all the information related to age, sex and ethnicity. All these details were mandatory with a view to ensure the statistical validity of the analysis.

### 4.2. Dataset Information

Considering the filters described in the previous section, we selected the dataset available in GEO with the ID GSE63060 (the same dataset is also available in the database ArrayExpress with the ID E-GEOD-63060) for the analysis. The dataset was included in research related to multi-tissue performed by Sood et al. [71], who deposited all the data in the databases previously reported. The dataset included either AD patients or subjects with MCI, as well as CTL samples with all the information about condition, ethnicity, age, and gender. The dataset GSE63060 includes 329 samples of which 104 were CTL, 80 MCI, and 145 AD. In the dataset, all the information related to the extraction protocol, label protocol, and hybridization protocol was also reported. Table 3 reports the mean age and sex distribution of the different samples across the 3 groups included in the dataset.

All the additional information related to each sample and all the probes analyzed are available in Supplementary Table S6. As reported from GEO dataset, all the RNA was extracted from blood, and the extraction process was performed following the manufacturer’s instructions, using the PAXgene™ Blood RNA Kit (Qiagen, Hilden, Germany). The labeling process was carried out using the TotalPrep™ RNA Amplification Kit (Ambion, Austin, TX, USA), using biotin as marker. Regarding hybridization, complementary DNA (cDNA) was synthesized from 200 ng of total RNA using the TotalPrep™ RNA Amplification Kit (Ambion). The described processes were followed by amplification, biotinylation of complementary RNA (cRNA), and hybridization to the microarray platform. The scanning process was conducted using an Illumina BeadArray Scanner (Illumina, San Diego, CA, USA), ensuring precise detection and quantification of hybridized RNA signals. Illumina HumanHT-12 V3.0 expression beadchip was used to obtain the transcriptomic data.

### 4.3. Bioinformatics Analysis

Non-normalized counts were downloaded along with information about the samples and probes. Raw data were manipulated using R v.4.2.2 (R Core Team), and the statistical analysis was performed using the functions included in the package limma v.3.54.2 [72] of Bioconductor v. 3.16 [73]. To address the potential technical variability between arrays, raw data were normalized. First, a log_2_ transformation was applied, followed by normalization using the “normalizeBetweenArrays” function. Specifically, we applied quantile normalization, a method robust against outliers, recommended for single-channel microarray data. In all comparisons between CTL and AD for both datasets, information related to gender, age, and ethnicity were considered as additional parameters to improve the quality of the analysis. In detail, we designed the matrix to be used by the model to fit each gene setting the variable “Condition” (CTL or MCI or AD) as factor and the variables “Gender”, “Age” and “Ethnicity” as covariates. The design matrix used to perform the different comparisons is available in Supplementary Table S7. Thus, the linear model that represents the change in the expression level of each gene is computed following Equation (1):

Expression = α1 · Condition + α2 · Gender + α3 · Age + α4 · Ethnicity.(1)

**Figure 5:**
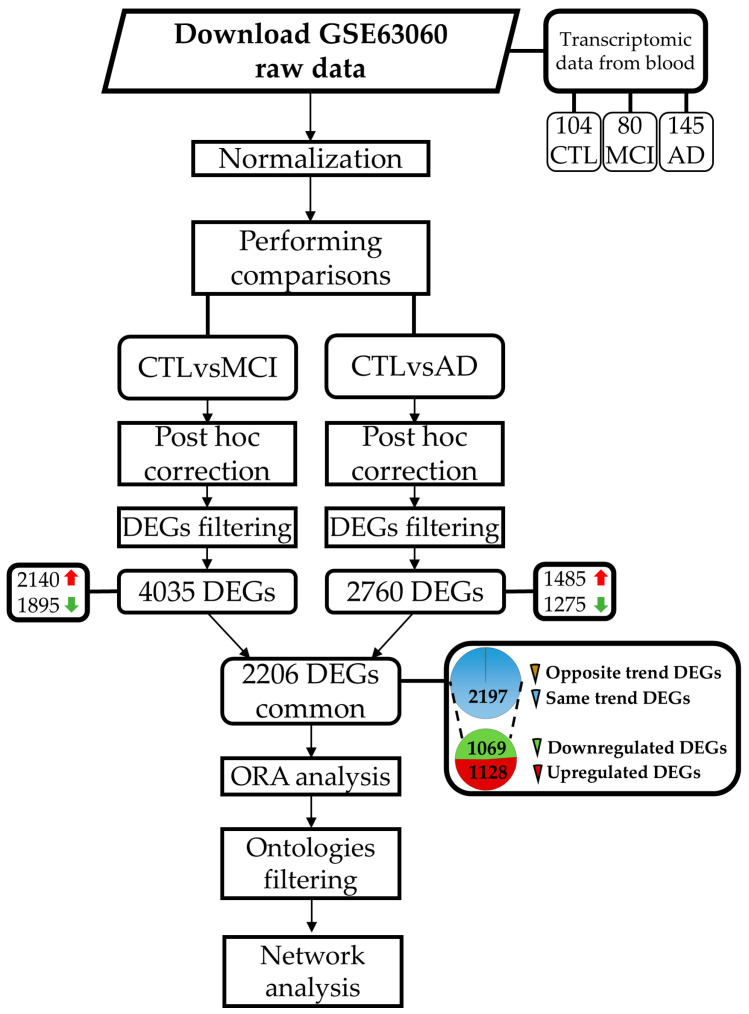
Workflow that summarizes all the steps followed to perform our analysis reported in the Discussion section. Red arrows refer to upregulated DEGs, whereas green arrows refer to downregulated DEGs.

Benjamini–Hochberg post hoc correction was used to adjust the *p*-value and reduce the number of false positive DEGs in our analysis. All the genes with a q-value < 0.05 were considered as statistically significant DEGs. The DEGs in both comparisons (CTLvsMCI and CTLvsAD) were used for ORA, performed using the package “clusterProfiler” v.4.6.2 [74]. This analysis confirmed that GO that showed statistically significant changes in both comparisons. A network analysis was also implemented to obtain an additional filter and to highlight the connections among the altered DEGs. To obtain all the connections among the DEGs resulting from the analysis, we used STRING v.12.0 [75], consulted on 1 June 2025. The information obtained from the STRING database was studied through the software “cytoscape” v.3.10.3 [76]. Figure 5 summarizes the workflow used for the analysis.

## 5. Conclusions

In this work, we investigated the early molecular changes associated with MCI and AD in peripheral blood samples. Our bioinformatics analysis revealed a consistent dysregulation of genes involved in ribosomal formation and biogenesis, as well as splicing processes. These pathways are essential for maintaining proper proteostasis and cellular function. Although it is known that such changes happen in the brain of AD patients, our results indicate that similar signals can be found in blood samples as early as the MCI stage. While further validation is needed, our findings highlight the potential of blood-based transcriptomic signatures as accessible indicators for early detection and intervention in AD.

## Figures and Tables

**Table 1:** Genes included in the gene families described in Figure 4.

Genes Family	Genes Included
EXOSC	*EXOSC1*, *EXOSC3*, *EXOSC7*, *EXOSC8*, *EXOSC9*
MRPL	*MRPL1*, *MRPL3*, *MRPL13*, *MRPL15*, *MRPL17*, *MRPL18*, *MRPL21*, *MRPL22*, *MRPL24*, *MRPL27*, *MRPL32*, *MRPL33*, *MRPL35*, *MRPL36*, *MRPL39*, *MRPL40*, *MRPL42*, *MRPL45*, *MRPL46*, *MRPL47*, *MRPL48*, *MRPL50*, *MRPL51*, *MRPL58*
MRPS	*MRPS7*, *MRPS17*, *MRPS18C*, *MRPS21*, *MRPS22*, *MRPS23*, *MRPS28*, *MRPS31*, *MRPS33*
RPL	*RPL3*, *RPL4*, *RPL5*, *RPL6*, *RPL7*, *RPL11*, *RPL12*, *RPL17*, *RPL21*, *RPL23*, *RPL24*, *RPL26*, *RPL26L1*, *RPL27*, *RPL30*, *RPL31*, *RPL34*, *RPL35*, *RPL35A*, *RPL36AL*, *RPL39*, *RPL41*
RPS	*RPS3A*, *RPS4X*, *RPS6*, *RPS7*, *RPS10*, *RPS12*, *RPS13*, *RPS14*, *RPS17*, *RPS18*, *RPS20*, *RPS21*, *RPS24*, *RPS25*, *RPS27*, *RPS27A*, *RPS27L*, *RPS29*

For each gene family listed in the first column, the corresponding genes are reported in the second column.

**Table 2:** Table summarizing DEG information.

DEGs	Regulation	Function	Possible Role in Neurodegeneration	References
*UTP6*, *UTP11*, *UTP14A*	Downregulated	Required for SSU biogenesis and involved in nucleolar processing of pre-18 S ribosomal RNA.	Downregulation of UTP11 is observed in early stages of AD and is implicated in nucleolar stress and altered ribosomal biogenesis.	[62]
*RPF1*, *RPF2*	Downregulated	Involved in ribosomal large subunit assembly.	Depletion of RPF2 blocks the 27 pre-RNA-processing process, inducing nucleolar stress.	[63]
*NOP58*, *NOP10*, *DKC1*	Downregulated	Required for 60 S ribosomal subunit biogenesis. Core component of snoRNP particles.	Depletion of DKC1 and NOP10 causes increased oxidative stress and impaired ribosomal biogenesis. Both of these processes are closely implicated in the pathophysiology of neurodegenerative diseases.	[64]
*RPL30*, *RPL34*, *RPL4*	Downregulated	Component of the large ribosomal subunit.	Downregulation of these ribosomal proteins are observed in hippocampal samples from AD.	[36]
*EIF3E*, *EIF3M*	Downregulated	Component of the eIF-3 complex, which is required for several steps in the initiation of protein synthesis.	The low expression of the factor eIF3 was observed in hippocampus samples from patients with AD.	[49]
*SNRPG*, *SNRPD2*, *SNRPF*, *SNRPA*, *SNRPB2*	Downregulated	Structural components of the protein core of the U1 snRNP complex, involved in the recognition of the 5′ splicing site.	There is no direct evidence of their downregulation in the neurodegeneration process. However, alteration of the U1 snRNP complex is associated with splicing defects in the brains of AD patients.	[65]
*PRPF18*, *ISY1*, *SLU7*	Downregulated	Factors involved in pre-RNA splicing	Involved in DNA splicing and repair (via APE1); although there is no direct evidence in neurodegeneration, its downregulation may reduce the response to genotoxic damage.	[66]
*GEMIN6*, *GEMIN2*	Downregulated	The SMN complex catalyzes the assembly of snRNPs, the components of spliceosome.	The SMN complex deficit may contribute to SMA and neuronal splicing dysfunctions and neurodegeneration.	[67]
*LTO1*	Upregulated	Required for biogenesis of the large ribosomal subunit and initiation of translation.	The upregulation, although not directly associated with neurodegeneration, could reflect a compensatory mechanism against oxidative stress, one of the mechanisms relevant in neurodegenerative diseases.	[40]

For the all the DEGs, reported in the first columns, we describe the possible role in neurodegeneration with their regulation in our analysis.

**Table 3:** Dataset GSE63060 information.

Sample Groups	Age Mean	Sex Distribution
CTL	72.4 ± 6.3	42 M 62 F
MCI	74.5 ± 6.0	41 M 39 F
AD	75.4 ± 6.6	46 M 99 F

For the 3 groups included in the dataset, we report the age mean with the standard error in the first column, followed by the sex distribution in absolute values reported in the second column.

## Data Availability

The data presented in this study are openly available in the Gene Expression Omnibus (GEO) database (https://www.ncbi.nlm.nih.gov/gds; accessed on 10 May 2025), under the accession number GSE63060.

## Supplementary Materials

The following supporting information can be downloaded at https://www.mdpi.com/article/10.3390/ijms26157303/s1.

## Abbreviations

The following abbreviations are used in this manuscript:

AD	Alzheimer’s disease
Aβ	Amyloid beta
FAD	AD familial form
SAD	AD sporadic form
MCI	Mild cognitive impairment
CTL	Healthy controls
CTLvsAD	Comparison among CTL and AD groups
CTLvsMCI	Comparison among CTL and MCI groups
DEGs	Differentially expressed genes
FDR	False discovery rate
FC	Fold change
GO	Gene ontology
ORA	Over-representation analysis
BP	Biological process
MF	Molecular function
CC	Cellular component
SSU	Small subunit
GEO	Gene expression omnibus
